# Central auditory test performance predicts future neurocognitive function in children living with and without HIV

**DOI:** 10.1038/s41598-024-52380-1

**Published:** 2024-02-01

**Authors:** Jeff Joseph, Christopher Niemczak, Jonathan Lichtenstein, Anastasiya Kobrina, Albert Magohe, Samantha Leigh, Christin Ealer, Abigail Fellows, Catherine Reike, Enica Massawe, Jiang Gui, Jay C. Buckey

**Affiliations:** 1 Department of Quantitative Biomedical Sciences, Geisel School of Medicine at Dartmouth, Hanover, NH USA; 2 Space Medicine Innovations Laboratory, Geisel School of Medicine at Dartmouth, Hanover, NH USA; 3 Department of Medicine, Dartmouth-Hitchcock Medical Center, Lebanon, NH USA; 4 Department of Psychiatry, Dartmouth-Hitchcock Medical Center, Lebanon, NH USA; 5 The Dartmouth Institute for Health Policy and Clinical Practice, Geisel School of Medicine at Dartmouth, Hanover, NH USA; 6 Muhimbili University of Health and Allied Sciences, Dar es Salaam, Tanzania; 7 Department of Biomedical Data Science, Geisel School of Medicine at Dartmouth, Hanover, NH USA

**Keywords:** Computational biology and bioinformatics, Developmental biology, Neurology

## Abstract

Tests of the brain’s ability to process complex sounds (central auditory tests) correlate with overall measures of neurocognitive performance. In the low- middle-income countries where resources to conduct detailed cognitive testing is limited, tests that assess the central auditory system may provide a novel and useful way to track neurocognitive performance. This could be particularly useful for children living with HIV (CLWH). To evaluate this, we administered central auditory tests to CLWH and children living without HIV and examined whether central auditory tests given early in a child’s life could predict later neurocognitive performance. We used a machine learning technique to incorporate factors known to affect performance on neurocognitive tests, such as education. The results show that central auditory tests are useful predictors of neurocognitive performance and perform as well or in some cases better than factors such as education. Central auditory tests may offer an objective way to track neurocognitive performance in CLWH.

## Introduction

Approximately 1.8 million children under age 15 are living with HIV (UNAIDS, 2020)^1^. Although antiretroviral treatment (ART) and HIV care have improved in the last 2 decades, children living with HIV (CLWH) experience a diverse set of comorbidities, including neurocognitive impairment^2–4^. HIV related neurocognitive disorder typically presents with executive dysfunction and memory impairment. Attention, multitasking, impulse control, and judgment are also disrupted^5^. CLWH perform significantly worse on planning, reasoning, processing speed, and visuospatial tasks relative to healthy controls^6–8^. Early detection of neurocognitive deficits in CLWH is critical because of the detrimental life-long effects these deficits can have on educational outcomes, employment, and relationships, especially in low- middle-income countries (LMICs) where most cases of HIV exist.

A variety of neurocognitive test batteries are used to assess cognitive impairment in adults and children. Neurocognitive batteries are time-consuming, labor intensive, and must be administered and interpreted by trained professionals. This makes their use problematic in LMICs, where such resources are limited^9^. Neurocognitive test results are also affected by an individual's socioeconomic and educational status^10^. Low socioeconomic status and poor educational opportunities in children are often associated with poor neurocognitive function, which is also associated with HIV infection^11,12^. Therefore, predicting who may develop neurocognitive deficits due to HIV can be challenging. Discerning whether poor neurocognitive function in children is HIV related or due to other environmental and social factors can be difficult.

A different approach to assessing brain function is to evaluate the brain’s ability to process complex sounds (i.e., central auditory processing). Tests assessing this ability, termed central auditory tests (CATs), include measures of the ability to understand speech in background noise, detect short periods of silence in a continuous noise presentation (gaps-in-noise), or deciphering different words presented to both ears simultaneously. While peripheral auditory function (i.e., hearing sensitivity to tones) is necessary for accurate central auditory processing, CATs go beyond the peripheral auditory system and require rapid cortical processing, concentration, attention, and integration of executive function within the brain. Measures of central auditory processing correlate with neurocognitive function in people living with HIV^13^. Niemczak et al.^13^ showed that central auditory tests (CATs) are positively associated with both learning and working memory measures. Furthermore, recent research shows that electrophysiological measures of sound processing [e.g. Frequency Following Response (FFR)] can be used as markers of central nervous system dysfunction in CLWH (Ealer et al., Accepted at AIDS). These results suggest CATs might be useful for tracking or predicting neurocognitive function.

Because the interaction of multiple factors can affect neurocognitive function, a machine learning approach to prediction may be useful. Machine learning allows multiple factors to be considered to improve overall predictive abilities from a diverse data set. To understand whether CATs in addition to other factors such as education can predict neurocognitive function, machine learning models such as random forest (RF), eXtreme Gradient Boosting (XGBoost), and support vector machines (SVM) may provide an improved analytical method^14–16^. Machine learning models consistently yield better predictive performances than traditional statistical models with biomedical data or other data with high levels of complexity^17–19^. High predictability machine learning models can detect *predictor-outcome* dependencies that traditional statistical models may fail to detect. Furthermore, machine learning models are better suited to learn nonlinear trends and patterns in the longitudinal *predictor-outcome* relationship when hyperparameters (i.e., model settings) are appropriately tuned during model training. These models have not been previously used to assess the predictive ability of central auditory function for future cognitive development in children.

In this study, we examined whether CATs early in childhood could predict cognitive performance at a later time point. We used longitudinal CAT scores and *state-of-the-art* machine learning models to examine the predictive ability of CATs for neurocognitive deficits in children living with and without HIV. Specifically, we investigated the ability of CAT performance to predict performance on Leiter-3 neurocognitive test composites (*Nonverbal IQ, Processing Speed,* and *Nonverbal Attention/Memory*) administered 0.5–1.5 years later. We used longitudinal data collected from children in Dar es Salaam, Tanzania. We hypothesized that predictors involving CATs would yield useful predictions of subsequent Leiter-3 composite performance.

## Results

### Predicting neurocognitive function

We used data from children between the ages of 3–10 years who were part of a longitudinal study of HIV and neurocognitive performance in Dar es Salaam, Tanzania. Children came at 6-month to 1-year intervals to have measurements of both central auditory function using CATs and cognitive performance using the Leiter-3. CATs could be either behavioral (the child heard a sound and gave a response) or electrophysiological (electrical activity of populations of neurons in the auditory pathway evoked by an acoustic stimulus). Children with less than three visits and/or missing CATs and Leiter-3 scores were excluded from the analysis. We used 7 different sets of variables to predict neurocognitive function as measured by the Leiter-3 (see “Methods” section for details). The performance metrics used were area under the receiver operating curve (AUROC/AUC) and F1-score. Rather than looking at model performance at various sensitivity and specificity thresholds, the AUC provides a single summary measure of a model’s overall predictive ability across all possible sensitivity and specificity thresholds. Accuracy is often used when there is balance in the distribution of outcome classes and it is simply the proportion of correct predictions. In our case a class imbalance exists, therefore it is more statistically appropriate to use F1-score as a model performance metric. The F1-score accounts for the fact that the minority class (class with smaller sample) may be harder to detect than the majority class. Consequently, it provides a more balanced measure of a model’s performance across all classes than accuracy does in the presence of class imbalance. Both metrics are presented on a 0–1 scale with higher values (closer to 1) being best. Four machine learning models were used: Random forest, eXtreme Gradient Boosting, Logistic regression, and Support Vector Machines.

CATs demonstrated predictive ability for 2 of the 3 Leiter composites. Machine learning models using CATs as predictors achieved the highest predictive capabilities. For the *Nonverbal IQ* Leiter-3 composite, models that contained behavioral CATs alone (F1 = 0.79, AUC = 0.73), electrophysiological CATs and covariates (e.g. education) (F1 = 0.65, AUC = 0.72), and behavioral and electrophysiological CATs and covariates (F-1 = 0.75, AUC = 0.74) performed best. Models for the Leiter-3 *Processing Speed* composite performed best when behavioral CATs (F1 = 0.64, AUC = 0.76) and behavioral and electrophysiological CATs (F1 = 0.84, AUC = 0.85) were included as predictors. All model and predictor combinations failed to reach AUC > 0.70 for *Nonverbal Attention/Memory* composite (see Tables 1, 2, 3, 4 for full results).

**Table 1:** Summary of distribution of demographic and central auditory tests data for HIV+ and HIV− children.

	HIV−	HIV+	*p* value
Female	36	17	
Male	36	20	> 0.05
	Mean (SD)	Mean (SD)	
Maximum education (years)	2.605 (1.596)	2.716 (1.498)	> 0.05
Hearing in noise test (HINT)	− 7.664 (3.252)	− 6.753 (3.384)	> 0.05
Triple digit test (TDT)	− 21.681 (6.210)	− 21.105 (6.912)	> 0.05
Staggered spondaic words test (SSW)	17.580 (15.436)	15.743 (15.672)	> 0.05
ABR wave V amplitude (slow click)	0.208 (0.092)	0.187 (0.075)	> 0.05
ABR wave V latency (slow click)	5.835 (0.291)	5.857 (0.423)	> 0.05
ABR wave V amplitude (fast click)	0.155 (0.059)	0.141 (0.063)	> 0.05
ARR wave V latency (fast click)	6.092 (0.361)	6.058 (0.364)	> 0.05
FFR fundamental frequency (F0)	0.004 (0.002)	0.004 (0.003)	> 0.05
FFR first formant (F1)	0.009 (0.006)	0.010 (0.009)	> 0.05
FFR signal to response correlation	0.180 (0.058)	0.186 (0.068)	> 0.05
FFR response consistency	0.660 (0.238)	0.624 (0.269)	> 0.05

The first *p* value assesses the distribution of gender across HIV groups while the remaining is the result comparing the mean of each variable by HIV group.

**Table 2:** Results for predicting Nonverbal IQ.

Model variables	Models			
	Random forest	*eXtreme Gradient Boosting*	Logistic regression	Support vector machines
Education, gender, and HIV	0.73 0.57	0.68 0.52	0.72 0.67	0.75 0.69
CATs (behavioral)	0.68 0.52	0.79 0.73	0.62 0.47	0.65 0.53
CATs (electrophysiological)	0.67 0.59	0.76 0.66	0.70 0.66	0.68 0.69
CATs (behavioral and electrophysiological)	0.78 0.61	0.74 0.65	0.70 0.66	0.62 0.52
CATs (behavioral), education, gender, and HIV	0.75 0.60	0.70 0.54	0.65 0.62	0.72 0.63
CATs (electrophysiological), education, gender, and HIV	0.75 0.56	0.75 0.62	0.72 0.67	0.65 0.72
CATs, education, gender, and HIV	0.80 0.63	0.71 0.56	0.75 0.74	0.74 0.65

There were 24 children in the *below age-based expectations* category for the training set, and 8 children in the testing set. The top number in each box is the F1-score and the bottom number is the AUC.

**Table 3:** Results for predicting *Processing Speed.*

Model variables	Models			
	Random forest	*eXtreme Gradient Boosting*	Logistic regression	Support vector machines
Education, gender, and HIV	0.74 0.57	0.80 0.67	0.62 0.54	0.71 0.44
CATs (behavioral)	0.80 0.62	0.77 0.65	0.67 0.57	0.64 0.76
CATs (electrophysiological)	0.74 0.47	0.68 0.40	0.62 0.47	0.69 0.42
CATs (behavioral and electrophysiological)	0.75 0.48	0.73 0.50	0.64 0.37	0.85 0.84
CATs (behavioral), education, gender, and HIV	0.78 0.55	0.80 0.62	0.67 0.57	0.62 0.54
CATs (electrophysiological), education, gender, and HIV	0.71 0.44	0.69 0.47	0.62 0.47	0.68 0.52
CATs, education, gender, and HIV	0.75 0.48	0.71 0.49	0.66 0.39	0.71 0.60

There were 17 children in the *below age-based expectations* category for the training set, and 6 children in the testing set.

**Table 4:** Results for predicting *Nonverbal Attention/Memory.*

Model variables	Models			
	Random forest	*eXtreme Gradient Boosting*	Logistic regression	Support vector machines
Education, gender, and HIV	0.72 0.45	0.69 0.47	0.58 0.44	0.57 0.30
CATs (behavioral)	0.72 0.45	0.69 0.42	0.67 0.57	0.53 0.27
CATs (electrophysiological)	0.75 0.48	0.72 0.45	0.71 0.60	0.67 0.57
CATs (behavioral and electrophysiological)	0.71 0.44	0.78 0.55	0.74 0.69	0.64 0.49
CATs (behavioral), education, gender, and HIV	0.72 0.45	0.74 0.47	0.58 0.44	0.59 0.32
CATs (electrophysiological), education, gender, and HIV	0.74 0.47	0.73 0.50	0.68 0.52	0.62 0.47
CATs, education, gender, and HIV	0.78 0.55	0.77 0.53	0.66 0.50	0.68 0.40

There were 17 children in the *below age-based expectations* category for the training set, and 6 children in the testing set.

We used the arithmetic mean (m) and standard deviation (sd) of F1 and AUC scores across the four machine learning models (see “Methods” section) to determine which sets of predictors performed best and were most stable. For *Nonverbal IQ*, the models that included electrophysiological CATs and covariates as predictors yielded the highest and most stable predictive performances across the four models (m F1 = 0.72 ± 0.04, m AUC = 0.64 ± 0.06). For *Processing Speed*, the models that included the behavioral CATs yielded the highest and most stable results across the models (m F1 = 0.72 ± 0.07, m AUC = 0.65 ± 0.07).

## Discussion

As expected, factors such as education and HIV were useful for predicting performance on neurocognitive tests. The important finding, however, was that CATs alone were often the best predictor of subsequent poor neurocognitive function. This is interesting because CATs and the Leiter-3 differ substantially in how they engage the brain. The Leiter-3 is a totally non-verbal test. Instructions are given via gesture and facial expressions and no information is provided using speech. CATs, by contrast, test the underlying processes involved in processing speech and auditory signals. Nevertheless, CATs predict Leiter-3 performance suggesting they may both depend on the same underlying neurological processes.

The results also suggest CATs may be useful as an early predictor. CATs taken early in this longitudinal study predicted cognitive performance later on. While these results are preliminary, they present the possibility that CATs could be used early in a child’s development and perhaps tracked over time to obtain information on how HIV may be affecting the brain. By combining CAT results with environmental and social factors (including HIV and education) at young ages and using the predictive ability of readily available machine learning models we can reach better predictions of who may have lower neurocognitive performance at later ages in children with HIV. This is of high importance given the aforementioned findings that CLWH have higher risk of neurocognitive deficits. Implementing standardized testing at any age is challenging, particularly in LMICs. There are always cultural elements to consider, including the novelty of testing and the influence of normative differences between how tests are developed in the West and the cultural context in which they are being used. CATs can be completed with less influence from culture, while providing a strong measure of brain function hence they are crucial in improving predictions of neurocognitive performance in the LMCIs. Having tools that can predict functional problems in the future, and at such a young age, can lead to more rapid identification of developmental concerns and allow for earlier intervention to promote better outcomes^20^. This would be a significant improvement over trying to perform detailed neurocognitive testing.

A limitation of our approach is we observed instability in the performance of the machine learning models. For instance, performance varied from model to model for certain predictors (XGBoost − AUC = 0.73 to SVM − AUC = 0.53 for behavioral CATs only as predictors with *Nonverbal IQ* as the outcome). This is likely a consequence of limited sample size and/or low representation of the *below age-based expectation* class (the neurocognitive underperformers) in the testing set. With low representation of the *below age-based expectation* class in the testing set, a single misclassification or a correct classification is enough to significantly increase or decrease the predictive performance of a model. We also observed CATs did not perform well predicting the Nonverbal Memory composite performance. It is challenging to discern why that is the case. It may be due to the nature of the specific tasks in the Nonverbal Memory composite, which rely on higher-order processes (e.g., working memory, cognitive flexibility) that are less apparent in the Nonverbal IQ or Processing Speed composites. In this sense, CATs may align best with general cognitive reasoning and speed of information processing. Another challenging aspect of the analytical phase of our work is the low number of predictors in the data. Machine learning models reach their full predictive potential in the presence of a large number of predictors where they can find high order levels of interplay between those predictors. With a low number of predictors in the data we also run the risk of creating correlated trees in the random forest models which would cause overfitting of the models on the training set and subsequent underperformance on the testing set. Further investigation is needed on larger data to confirm our findings and obtain more consistent and stable predictive performances on our models. Additionally, we may need to follow children for a longer period of time to establish early CATs as true and meaningful predictors of later neurocognitive function.

In summary, we built machine learning models that used CATs and other demographics such as to predict neurocognitive function in CLWH and healthy controls with high accuracy. In multiple instances, models that contained CATs as predictors outperformed models that contained covariates only as predictors. We conclude that both behavioral and electrophysiological CATs have promise as predictors of neurocognitive function.

## Methods

### Participants and data

The data for CLWH and HIV-negative children were collected as part of an on-going longitudinal study in Dar es Salaam, Tanzania. Our research protocol was approved by the Committee for Protection of Human Subjects of Dartmouth College and the Research Ethics Committee of Muhimbili University of Health and Allied Sciences and all methodologies steps were conducted in accordance with the relevant guidelines and regulations.

Participants were recruited from local pediatric programs, district hospitals, and schools. Informed consent was obtained for all minors in this study from a parent and/or a legal guardian. As part of the study, participants attended bi-annual follow-ups up to age 6 and then annually after that. At each visit peripheral auditory function, CATs (behavioral and electrophysiological), and the Leiter-3 were collected.

#### Inclusion criteria

Data for 109 children were selected from a dataset using the following criteria. At the time of enrollment children were 3–11 years old, had normal hearing in both ears (i.e., ≤ 25 dB HL at 0.5, 1.0, 2.0, and 4.0 kHz), no history of exposure to traumatic noise, and normal tympanometry. Children with a history of mental illness, neurological disease, or loss of consciousness were excluded, as these factors impact performance on CATs^21–23^. HIV status was confirmed in children using medical records or a rapid HIV test and reconfirmed using an ELISA assay. Children with 3 or more visits were included in the analysis. Children with missing scores for more than one CAT, more than two electrophysiological CATs, or Leiter-3 were excluded from the analysis, as missing data can impact the predictive abilities of machine learning models, especially in small datasets^24^.

#### Audiometry

Pure-tone thresholds were collected in all children for 0.5, 1.0, 2.0, 4.0, 6.0, and 8.0 kHz using a Békésy-like tracking and Modified Hughson Westlake procedures (see Niemczak et al.^25^ for details). Thresholds of 25 dB HL or higher for each ear were considered abnormal. Pure tone average (PTA) was calculated by averaging thresholds from 0.5 to 4.0 kHz.

#### Behavioral central auditory tests

The Hearing in Noise Test (HINT), Triple Digit Test (TDT), and Staggered Spondaic Words Test (SSW) were used to measure central auditory processing in children (for details see Niemczak et al.^13^)^11^. HINT and TDT are used to assess one’s ability to perceive and process speech in noise, while SSW measures dichotic processing^26^. All tests were administered and presented in the Kiswahili language.

#### Electrophysiological tests

The acoustic brainstem response (ABR) followed a similar methodology to Niemczak et al.^25^. An Intelligent Hearing Systems SmartEP (Miami, FL) was used to record ABR measurements 100 μs rarefaction clicks presented at a rate of 21.1/s (slow) or 61.1/s (fast) at 80 dB sound pressure level to the right ear. The electrode montage consisted of the right earlobe as the inverting, ground at F_pz_, and the high forehead at F_z_ serving as the non-inverting electrode. Two repetitions of each click were recorded and averaged (total 2000 sweeps). Responses were filtered from 0.1 to 1.5 kHz. The absolute latencies and amplitudes of waves, I, III, and V were measured from baseline.

The frequency following response (FFR) was evoked using the /ba/ syllable, collected from all subjects using the same hardware as the ABR. The collection of the FFR has been described in-detail elsewhere^27^ and methodology follows Ealer et al. (in review). Stimuli were played monaurally to the right ear at 80 dB HL at a rate of 4.35 per second. Two runs of 3000 artifact-free responses were collected and responses were then offline filtered from 0.7 to 2 kHz. The /ba/ was primarily analyzed at the vowel region of the stimulus (i.e., the /a/), which is spectrotemporally static. The /ba/ stimulus was 180 ms in duration, and the vowel region was from 60 to 180 ms. The FFR was recorded with alternating polarity. When analyzing the FFR, we added these polarities together (added condition) or subtract them from one another (subtracted condition) to emphasize lower frequency information (i.e., fundamental frequency) or higher frequency information (i.e., formants), respectively.

#### Leiter-3

The Leiter International Performance Scales-Third Edition (Leiter-3). The Leiter-3 assesses neurocognitive functioning in children and adults from 3 to 75 years of age. Domains measured include fluid and categorical reasoning, visual identification, and mental sequencing. The test is entirely nonverbal, with instructions delivered via gestures and pantomime. Participants provide responses via pointing, block or manipulative placement, and paper-and-pencil task completion. We have demonstrated the feasibility and acceptability of the Leiter-3 in Tanzania^6,28^ (see Lichtenstein et al.^6^ for a discussion of training methods and procedures.)

#### Predictors

Predictors were extracted from early visits of all participants (see Table 1). We defined *early* as the visits prior to or at the median age of a child during their participation in the study. Gender, HIV status, the maximum number of years of education (i.e., years of education hereafter), and the best score for each behavioral and electrophysiological CAT during early visits were extracted. We defined gender, HIV, and years of education as covariates. We reason that maximum years of education and best CATs’ scores should be used to predict best performance on the Leiter-3 composites. Age was excluded as a variable as it is highly correlated with years of education (r = 0.83). Including age would introduce correlation bias to trees-based models^29^. Using our predictors, we constructed 7 models with a variety of variable combinations: (1) covariates alone, (2) behavioral CATs, (3) electrophysiological CATs, (4) behavioral and electrophysiological CATs, (5) covariates and behavioral CATs, (6) covariates and electrophysiological CATs, and (7) covariates with behavioral and electrophysiological CATs.

#### Outcome classes

Outcome variables were extracted for later visits. *Later* visits are defined as after the median age of a child during their participation in the study. Highest age-adjusted Leiter-3 scores (as opposed to raw scores) were extracted for three Leiter-3 composites (*Nonverbal IQ, Processing Speed,* and *Nonverbal Attention/Memory*)^30^. We divided the Leiter-3 scores by the age of the child at the time the test was administered. This accounts for the fact that older children should in reality outperform their younger counterparts. The scores were then converted into categorical neurocognitive outcome classes (i.e., *within age-based expectations* and *below age-based expectations*). A child’s neurocognitive function class assignment was based on where their Leiter-3 composite score fell on the overall distribution of Leiter-3 scores. For each Leiter-3 composite, a child with a score of 1 standard deviation below the mean was assigned to the *below age-based expectations* class (*class 1*), all other children were categorized as *within age-based expectations* (*class 0*). We used a 1 SD cutoff instead of 1.5 SD below the mean due to a limiting size of our sample. Only up to 5 out of 109 children had scores ≤ 1.5 SD below the mean for each Leiter-3 composite and models tend to underperform with such low representation of any class^31^.

### Predicting outcome classes

#### Models and performance metrics

We selected three widely used machine learning models (RF, XGBoost, and SVM) appropriate for the dimensions of our data. We assessed the predictive ability of a more traditional statistical model with logistic regression. All analytical steps were conducted in Python using the Scikit-learn package^32^. The selected tree-based models typically perform well when used with small datasets and SVM as well^33^.

After assigning each child to a neurocognitive function category based on their adjusted Leiter-3 scores for each composite, we randomly assigned 2/3 s of the data to the training set and the remaining 1/3 to the testing set while keeping a 2:1 ratio of *below age-based expectations* in training and testing sets respectively. We used the training set to find the best hyperparameters and the testing set for model evaluation. The final sample of children classified as *below age-based expectations* was 24 in *Nonverbal* 4.2.2 IQ, 17 in Processing Speed, and 17 for Nonverbal Memory.

We used performance metrics that account for the observed class imbalance in our data. The *F-measure* is a metric of choice in the machine learning literature when faced with class imbalances as it can assign different weights to precision and recall based on their relative contextual importance (see Eq. (1)):^32^

1 $$F = \left( {\beta^{2} + 1.0} \right) \times \left( {P\times R} \right) / (\beta^{2} \times P + R).$$

$\beta$ is a non-negative parameter striking the balance of relative importance between $P$ (precision) and $R$ (recall)^34^. Precision is the proportion of correct predictions out of all data points assigned to a class. On the other hand, recall is the proportion of data points from a given class correctly assigned to that class. In our case, we assigned $\beta$ to be equal to 1 indicating equal importance yielding:

2 $$F = 2\times P \times R / \left( { P + R} \right).$$

We also present the Area Under the Receiver Operating Curve (AUROC/AUC) for the models of interest as a second metric of assessing model performance.

#### Class balancing technique

Due to class imbalance in our data, the models would underperform in detecting the *below age-based expectations* (class 1) in the testing sets. Our testing sets were composed of 8 children in *Nonverbal IQ,* 6 in *Processing Speed,* and 6 in *Nonverbal Memory* Leiter-3 composites. To account for the imbalance, we set the class weight parameter of the models as *balanced* during model training, which was used to scale the loss function of each model during training. While training on each point, the error was multiplied by the weight assigned to the class of that training point, forcing the model to reduce its loss function by disproportionately penalizing each misclassification of the heavily-weighted (*below age-based expectations*: class 1) training points.

## Data Availability

All data generated or analyzed during this study are available from the author upon reasonable request.

## References

[1] Stover J, Glaubius R, Kassanjee R, Dugdale CM (2021). Updates to the spectrum/AIM model for the UNAIDS 2020 HIV estimates. J. Int. AIDS Soc..

[2] Heaton RK (2011). HIV-associated neurocognitive disorders before and during the era of combination antiretroviral therapy: Differences in rates, nature, and predictors. J. Neurovirol..

[3] Pourcher V, Gourmelen J, Bureau I, Bouee S (2020). Comorbidities in people living with HIV: An epidemiologic and economic analysis using a claims database in France. PLoS ONE.

[4] Zhan Y (2018). Speech in noise perception as a marker of cognitive impairment in HIV infection. Ear Hear.

[5] Saylor D (2016). HIV-associated neurocognitive disorder–pathogenesis and prospects for treatment. Nat. Rev. Neurol..

[6] Lichtenstein J (2022). Nonverbal cognitive assessment of children in Tanzania with and without HIV. Child Neuropsychol..

[7] Boivin MJ (2020). Early childhood development caregiver training and neurocognition of HIV-exposed Ugandan siblings. J. Dev. Behav. Pediatr..

[8] Laughton B, Cornell M, Boivin M, Van Rie A (2013). Neurodevelopment in perinatally HIV-infected children: A concern for adolescence. J. Int. AIDS Soc..

[9] Kammerer, B., Isquith, P. K. & Lundy, S. Approaches to assessment of very young children in Africa in the context of HIV. *Neuropsychol. Child. Africa Perspect. Risk Resil.* 17–36 (2013).

[10] Hackman DA, Farah MJ (2009). Socioeconomic status and the developing brain. Trends Cogn. Sci..

[11] Laughton B (2013). Neurodevelopment in perinatally HIV-infected children: A concern for adolescence. J. Int. AIDS Soc..

[12] Boivin MJ (2020). African multi-site 2-year neuropsychological study of school-age children perinatally infected, exposed, and unexposed to human immunodeficiency virus. Clin. Infect. Dis. Off. Publ. Infect. Dis. Soc. Am..

[13] Niemczak CE (2021). The relationship between central auditory tests and neurocognitive domains in adults living with HIV. Front. Neurosci..

[14] Breiman L (2001). Random forests. Mach. Learn..

[15] Chen L, Zheng D, Liu B, Yang J, Jin Q (2016). VFDB 2016: Hierarchical and refined dataset for big data analysis—10 years on. Nucleic Acids Res..

[16] Cortes C, Vapnik V (1995). Support-vector networks. Mach. Learn..

[17] Singal AG (2013). Machine learning algorithms outperform conventional regression models in predicting development of hepatocellular carcinoma. Am. J. Gastroenterol..

[18] Wong KK, Fienup DM, Richling SM, Keen A, Mackay K (2022). Systematic review of acquisition mastery criteria and statistical analysis of associations with response maintenance and generalization. Behav. Interv..

[19] Liew BX, Kovacs FM, Rügamer D, Royuela A (2022). Machine learning versus logistic regression for prognostic modelling in individuals with non-specific neck pain. Eur. Spine J..

[20] Wedderburn CJ (2019). Neurodevelopment of HIV-exposed uninfected children in South Africa: Outcomes from an observational birth cohort study. Lancet Child Adolesc Health.

[21] Margari L (2018). Non-verbal cognitive abilities in children and adolescents affected by migraine and tension-type headache: An observational study using the leiter-3. Front. Neurol..

[22] Divenyi PL, Haupt KM (1997). Audiological correlates of speech understanding deficits in elderly listeners with mild-to-moderate hearing loss. III. Factor representation. Ear Hear.

[23] Musiek FE, Baran JA, Pinheiro ML (1990). Duration pattern recognition in normal subjects and patients with cerebral and cochlear lesions. Audiology.

[24] Emmanuel T (2021). A survey on missing data in machine learning. J. Big Data.

[25] Niemczak CE (2023). Peripheral auditory function in Tanzanian children living with HIV with clinically normal hearing. JAMA Netw. Open.

[26] Katz J, Smith PS (1991). The staggered spondaic word test. A ten-minute look at the central nervous system through the ears. Ann. N. Y. Acad. Sci..

[27] White-Schwoch T (2020). Auditory neurophysiology reveals central nervous system dysfunction in HIV-infected individuals. Clin. Neurophysiol..

[28] Bonacina, S. *et al.* Pre-literacy assessment in children living with HIV in Tanzania: Comparison to results from children living without HIV in Tanzania and the US. *AIDS (London, England)* (2023). 10.1097/QAD.0000000000003529 PMC10164069 36928339

[29] Rabinowicz A, Rosset S (2022). Tree-based models for correlated data. J. Mach. Learn. Res..

[30] Roid, G. H. & Miller, L. J. *Leiter International Performance Scale-Revised (Leiter-R)*, vol. 10 (Stoelting, Wood Dale, IL, 1997).

[31] Jak AJ (2009). Quantification of five neuropsychological approaches to defining mild cognitive impairment. Am. J. Geriatr. Psychiatry.

[32] Pedregosa F (2011). Scikit-learn: Machine learning in Python. J. Mach. Learn. Res..

[33] Jiang J (2020). Boosting tree-assisted multitask deep learning for small scientific datasets. J. Chem. Inf. Model..

[34] Chinchor, N. & Sundheim, B. M. In *Fifth Message Understanding Conference (MUC-5): Proceedings of a Conference Held in Baltimore, Maryland, August 25–27, 1993.*

